# Controlling the Internal Structures of Polymeric Microspheres via the Introduction of a Water-Soluble Organic Solvent

**DOI:** 10.3390/polym10070789

**Published:** 2018-07-18

**Authors:** Yanping He, Xin Li, Tianci Zhu, Mengxing Shan, Linhua Zhu, Tian Si, Hong Wang, Yanlin Sun

**Affiliations:** 1 School of Chemical Engineering, Kunming University of Science and Technology, Chenggong Campus, Kunming 650504, China; grace.he1985@hotmail.com (Y.H.); 15914857399@163.com (X.L.); 18487152221@163.com (T.Z.); m18213910776@163.com (M.S.); hualing67731@126.com (L.Z.); jayzhou-521@163.com (T.S.); 2 Faculty of Science, Kunming University of Science and Technology, Chenggong Campus, Kunming 650504, China; kglxy@kmust.edu.cn

**Keywords:** microsphere, polymer, hollow, porous, solvent evaporation, water-soluble organic solvent

## Abstract

Polymeric microspheres with different internal structures have been widely used because of their characteristics in the structures. This paper reports a method of controlling the internal structures of polymeric microspheres via the introduction of a water-soluble organic solvent to the continuous phase in the foam phase preparation of porous polymeric microspheres. The introduction of a water-soluble organic solvent enables the control of polymeric microspheres’ internal structures, from porous to hollow. Because a water-soluble organic solvent is introduced, the organic solvent may be diffused toward the interface because of the affinity between the organic solvent and the oil droplets, resulting an accumulation of organic solvent molecules at the interface to form an organic solvent layer. The presence of this layer may decrease the evaporation rate of the internal organic solvent in an oil droplet, which extends the time for the mingling of porogen droplets to form a few large pores or even an extremely large single pore inside. This method is also capable of altering the thickness of hollow microspheres’ shells in a desired way, with improved efficiency, yield and the capacity for continuous use on an industrial scale.

## 1. Introduction

Polymeric microspheres with various internal structures have attracted increasing interest in modern science and technology due to their attractive characteristics, especially the usefulness of their inner spaces [1,2,3,4,5]. Considerable effort has been made to control the internal structures of polymeric microspheres, including the utilization of microbubbles as templates [6,7] or the template removal method [8], emulsion solvent extraction [9], evaporation [10] and diffusion method [11], the self-assembly method [12,13] and the double emulsion method [14]. However, the reported methods are not versatile as it is not easy to control polymeric microspheres’ internal structures by using a particular method. Only a few papers have reported controlling microsphere structures, from the porous to the hollow, by decreasing the polymer concentration [10,15]. Therefore, there is a need to develop a versatile method which can easily control the internal structures of polymeric microspheres. In our previous work [15], we demonstrated a time-saving method with a high yield for producing porous polymeric microspheres in a foam phase, which can be developed as a continuous process for industrial production. In this work, we are proposing a new strategy to control the internal structures of polymeric microspheres based on the reported method [15] by introducing a water-soluble organic solvent. It enables the easy control of the internal structures of polymeric microspheres; in particular, hollow structured polymeric microspheres can be prepared even at a very high concentration of polymer, and their thickness can be well controlled by varying polymer concentrations. The proposed method provides a practical industrial solution to make versatile polymeric microspheres.

## 2. Materials and Methods

Polymeric microspheres were prepared in a foam phase. Polystyrene (PS, polymer), n-heptane (porogen) and dichloromethane (DCM, an organic volatile solvent), and ethanol (EtOH, a water-soluble organic solvent) were supplied by Xi Long Chemical Co., Ltd., Nanjing, China (AR). Emulsion stabilizer polyvinyl alcohol 1788 (PVA) was supplied by Chengdu Kelon Chemical Regent Co., Ltd., Chengdu, China (AR). In a typical experiment, a mixture of PS, n-heptane and DCM was decanted into a 250 mL three-neck round-bottom flask, where the mixture was admixed with an aqueous solution containing PVA at a concentration of 1.0 wt % and EtOH at various concentrations. Experimental reagents are shown in Table 1. The resulting dispersion was immersed in a water bath at room temperature and thoroughly vortexed at 500 rpm for 15 min. After that, the temperature of a water bath was ramped up at 1 °C/min and kept at 38 °C for 30 min. The bubble phase thus generated, along with PS microspheres, was transferred to a beaker in which a magnetic bar stirred the liquid at 500 rpm. At the same time, hot water at 90 °C was continuously flown into the beaker and interacted thoroughly with the bubbles. The heated water allowed DCM to evaporate and removed n-heptane. An optical microscope (LEICA DM4000 M, Leica Microsystems, Shanghai, China and a scanning electron microscope (SEM, JEOL JSM-6500, Peabody, MA, USA) were used to detect and display the surface morphology and the internal structures of polymeric microspheres. The particle size of PS microspheres was measured by using a laser particle diameter analyzer (Microtrac S3500, York, PA, USA). Each sample was measured three times to calculate the average diameter and the standard error.

## 3. Results and Discussion

### 3.1. The Effect of Introducing a Water-Soluble Organic Solvent on the Internal Structures of Polymeric Microspheres

**Figure 1:**
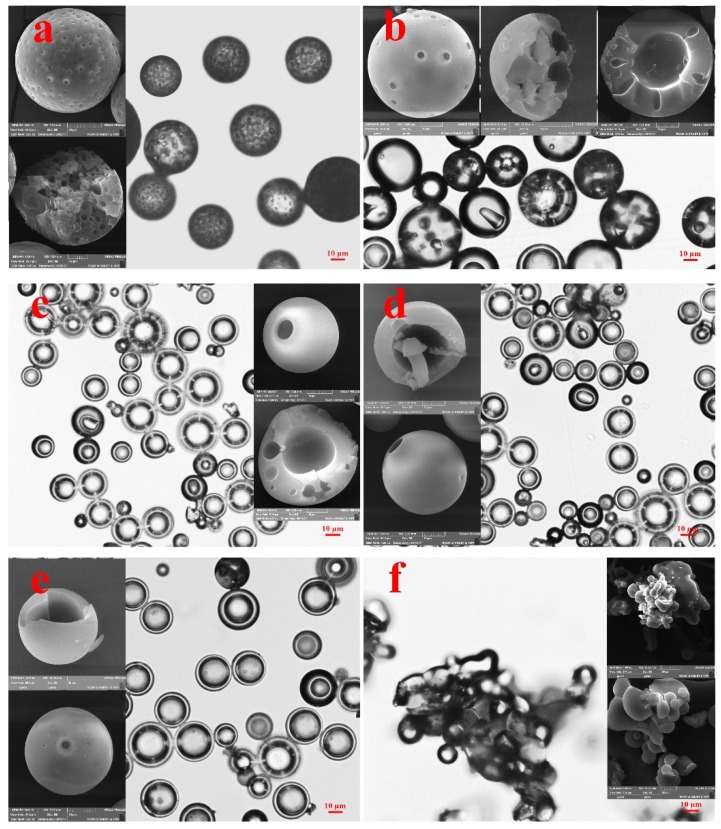
Optical microscope images and SEM images of PS microspheres prepared under varying experimental conditions: concentrations of EtOH at (**a**) 0, (**b**) 5.0 wt %, (**c**) 7.0 wt %, (**d**) 10.0 wt %, (**e**) 14.0 wt %, (**f**) 20.0 wt %.

**Figure 2:**
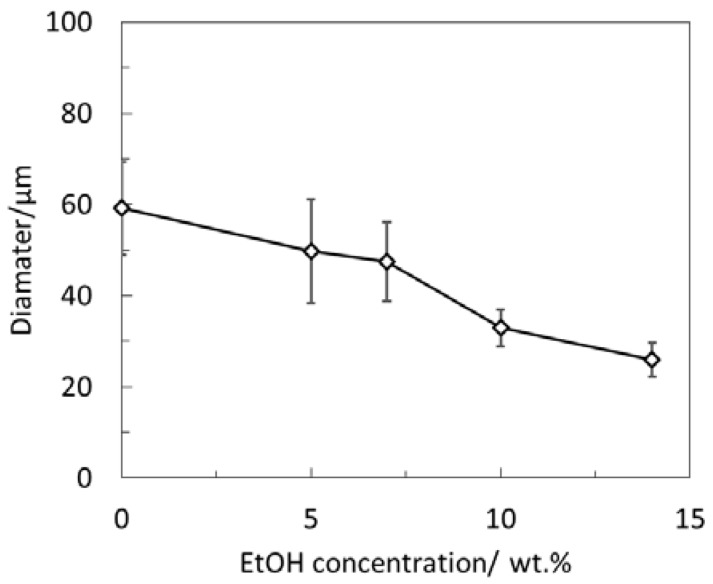
The diameter of PS microspheres as a function of the EtOH concentration.

The effect of introducing a water-soluble organic solvent (EtOH) to the continuous phase on the evolution of the internal structures of polymeric microspheres was first validated, as shown in Figure 1. Without EtOH, PS microspheres were in possession of porous structures, as shown in Figure 1a, which was the same as the results reported previously [15]. After introducing EtOH, the internal structures of PS microspheres evolved from porous to hollow. When EtOH concentration was low (5.0 wt %), both porous microspheres and hollow microspheres with a porous shell were observed (Figure 1b). However, the dimension of the pores was much larger than that prepared without EtOH. This was regarded as a transition point, as hollow structures were prepared above the concentration, as seen in Figure 1c–e. A further increase in EtOH concentration (20.0 wt %) led to the collapse of the structures. The result indicates that introducing a water-soluble organic solvent enables the control of the internal structures of polymeric microspheres. In addition, introducing EtOH also decreased the particle size of the prepared polymeric microspheres, as shown in Figure 2, which was attributed to the decrease in the surface tension caused by EtOH.

### 3.2. The Mechanism Resulting in Controlling the Internal Structures of Polymeric Microspheres

**Figure 3:**
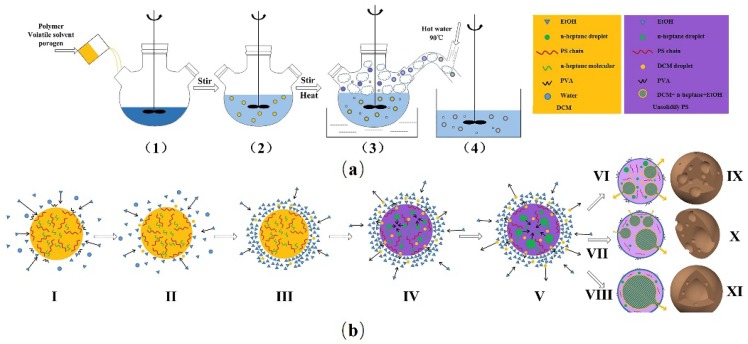
(**a**) Schematic diagrams of the process to prepare polymeric microspheres; (**b**) the mechanism to control the inner structures of polymeric microspheres by introducing a water-soluble organic solvent.

According to the above analysis, a probable mechanism to control the internal structures of polymeric microspheres has been deduced as shown in Figure 3. The solubility parameters of polystyrene, n-heptane and dichloromethane, ethanol, and water are 8.7–9.1, 7.45, 9.7, 12.7, 23.2 (cal^1/2^·cm^−3/2^) respectively [16] (pp. 52–54). EtOH molecules are more likely to diffuse towards the oil droplets (Figure 3I–III) during the suspension process (Figure 3(1–2)), which contain PS, n-heptane and DCM. Meanwhile, DCM molecules on the surface of the oil droplets diffuse towards EtOH molecules. EtOH and DCM are miscible and they may form a miscible organic solvents layer at the interface, as shown in Figure 3III. By elevating the temperature (Figure 3(3)), DCM molecules evaporate from the organic solvents layer, and then the organic solvents layer is inclined to extract DCM molecules form the out surface of an oil droplet as supplements, which drive the phase separation between PS and n-heptane (Figure 3IV). More importantly, the presence of the organic solvents layer acts as a barrier, and DCM may not evaporate completely instantaneously. PS is in a softening state with residual DCM, so both PS and n-heptane are flowing and they can easy mingle to form a few large pores or even one single extremely large pore in the inner of an un-solidified microsphere (Figure 3VI–VIII). When these un-solidified microspheres are delivered to the foam phase (Figure 3(3)), the organic solvents layer vanishes quickly and the residual DCM evaporates completely. Solidified PS microspheres (Figure 3IX–XI) are obtained after using hot water to remove n-heptane, PVA and the foam phase (Figure 3(4)). No miscible organic solvents layer as a barrier exists when only water is used as the continuous phase; the evaporation of DCM is too quick, which drives a quick phase separation between PS and n-heptane to form porous structures. When EtOH concentration is low, the barrier presents itself; however, the effect is weak. Therefore, porous structures are present, but the pore number decreases while the dimension increases, as illustrated in Figure 3IX. A further increase in EtOH concentration increases the thickness of the miscible organic solvents layer and the barrier effect increases. The softening PS and n-heptane have more time for mingling to form a few single large pores (Figure 3X) or even one single large pore (Figure 3IX) at a proper EtOH concentration. When EtOH concentration is extremely high, the organic solvents layer is thick enough to dissolve the spherical structure. No microsphere can be prepared. It is worthwhile to note that, after introducing EtOH, only a few or even one singe pore is observed on the microsphere surface. This is because the un-solidified microsphere is surrounded by the miscible organic solvents layer and the layer can dissolve polymers nearby, creating a smooth surface. After they are delivered to the foam phase, the residual DCM flows rush out from the most vulnerable areas to produce a few or even only one singe pore on the surface. 

### 3.3. Controlling the Thickness of Hollow Polymeric Microsphere Shells

**Figure 4:**
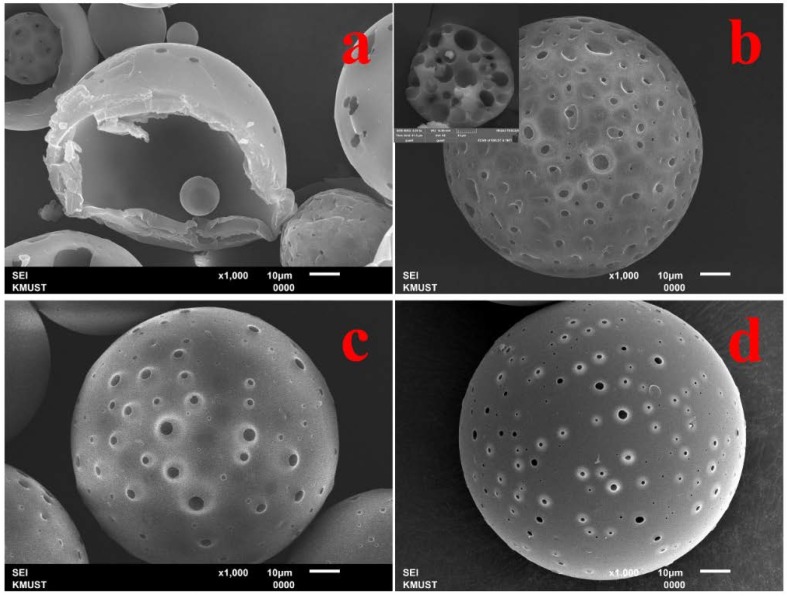

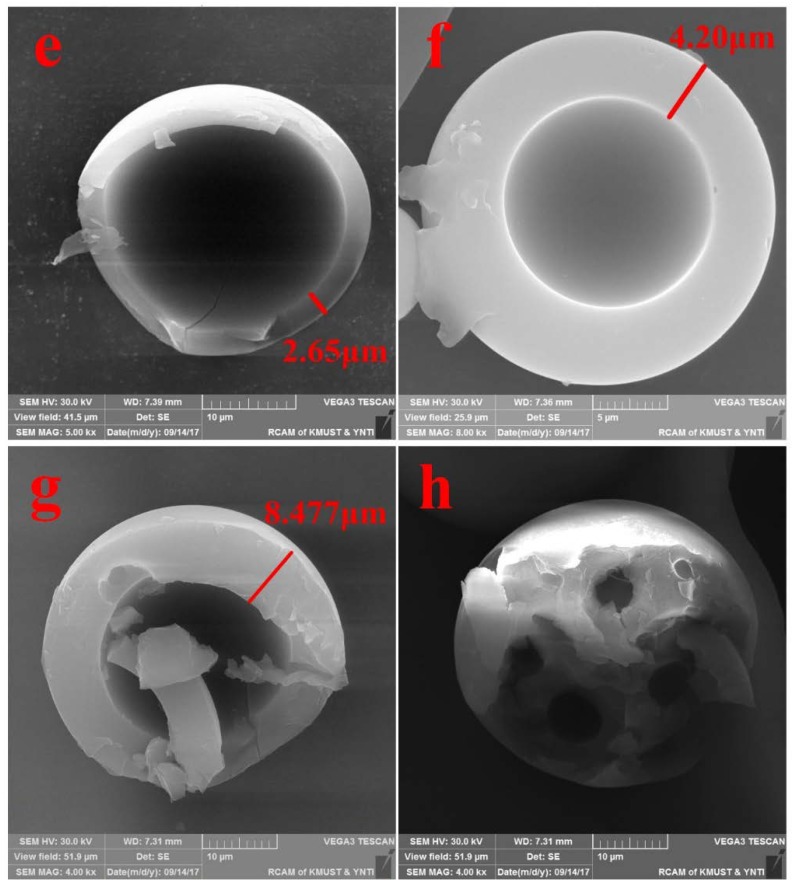
SEM images of PS microspheres prepared under varying experimental conditions: (**a**–**d**) water is used as a continuous phase and PS/DCM is 5 wt %, 10 wt %, 16.7 wt %, 25 wt %, respectively; (**e**–**h**) 10.0 wt % EtOH solution is used as a continuous phase and PS/DCM is 5 wt %, 10 wt %, 16.7 wt %, 25 wt %, respectively.

Introducing a water-soluble organic solvent leads to the formation of a miscible organic solvents layer at the interface as a barrier to decreasing the evaporation rate of the inner organic solvent, which alters the phase separation process. Therefore, it is supposed that hollow structures are much less reliant on the polymer concentrations, which is examined in Figure 4. As can be seen, without EtOH, the hollow structure was prepared only at 5.0 wt % (Figure 4a); after introducing 10 wt % of EtOH to the continuous phase, hollow structures were prepared when the polymer concentration was up to 16.7 wt % (Figure 4e–g). In addition, the thickness of the hollow microsphere shell is dependent on the polymer concentration (Figure 4e–g). Therefore, other than controlling the inner structures of polymeric microspheres, the thickness of the hollow microsphere shell can be altered by using this strategy. Compared with the method of preparing porous polymeric microspheres in a foam phase, which we reported before [15], only a water-soluble organic solvent is introduced to the new strategy. Therefore, the advantages of improved efficiency, high yield, and being able to work continuously on an industrial scale are also applicable. 

## 4. Conclusions

In this study, we demonstrated a new strategy to control the inner structures of polymeric microspheres. This enables the versatile control of the polymeric microspheres’ internal structures, from porous to hollow, only by introducing a water-soluble organic solvent to our previously reported method. This method is also capable of altering the thickness of the hollow microsphere shell in a desired way, with improved efficiency, yield and the capacity for continuous use on an industrial scale.

## Figures and Tables

**Table 1:** Preparative conditions of polymeric microspheres. PS: polystyrene; DCM: dichloromethane; PVA: polyvinyl alcohol.

Sample	PS g	n-Heptane g	DCM g	PVA g	Aqueous Phase 100.0 g	Temperature Gradient °C/min
E0	10.0	6.0	60.0	1.0	Water	1.0
E1	10.0	6.0	60.0	1.0	5.0 wt % EtOH solution	1.0
E2	10.0	6.0	60.0	1.0	7.0 wt % EtOH solution	1.0
E3	10.0	6.0	60.0	1.0	10.0 wt % EtOH solution	1.0
E4	10.0	6.0	60.0	1.0	14.0 wt % EtOH solution	1.0
E5	10.0	6.0	60.0	1.0	20.0 wt % EtOH solution	1.0
T1	3.0	6.0	60.0	1.0	10.0 wt % EtOH solution	1.0
T2	6.0	6.0	60.0	1.0	10.0 wt % EtOH solution	1.0
T3	10.0	6.0	60.0	1.0	10.0 wt % EtOH solution	1.0
T4	15.0	6.0	60.0	1.0	10.0 wt % EtOH solution	1.0
P1	3.0	6.0	60.0	1.0	Water	1.0
P2	6.0	6.0	60.0	1.0	Water	1.0
P3	10.0	6.0	60.0	1.0	Water	1.0
P4	15.0	6.0	60.0	1.0	Water	1.0
